# Marketing Organic Food from Millennials’ Perspective: A Multi-Theoretical Approach

**DOI:** 10.3390/foods11182721

**Published:** 2022-09-06

**Authors:** Booi Chen Tan, Suk Min Pang, Teck Chai Lau

**Affiliations:** 1 Faculty of Management, Multimedia University, Persiaran Multimedia, Cyberjaya 63100, Malaysia; 2 Ministry of Health Malaysia, Block E7, Complex E, Federal Government Administrative Centre, Putrajaya 62590, Malaysia; 3 Faculty of Business and Law, School of Marketing and Management, Taylor’s University, 1, Jalan Taylors, Subang Jaya 47500, Malaysia

**Keywords:** Millennials, organic food, purchase intention, socio-demographic, sustainability

## Abstract

This study examines the significant differences between the socio-demographic characteristics of Millennials in Malaysia and their intention to purchase organic food. In addition, the study also investigates the factors that influenced their purchase intention using a multi theoretical approach based on the Theory of Planned Behavior and Protection Motivation Theory. A questionnaire-based approach was applied. Data were collected via a face-to-face method at organic and non-organic food shops located in Klang Valley, Malaysia. SPSS and PLS-SEM were used to analyze 214 useable samples. The results from the independent sample T-test and ANOVA test showed that there were no significant differences between gender, age, marital status, educational level and ethnicity and intention to purchase organic food; while occupation, monthly income and prior purchase experience were found to have differences on this intention. In addition, a structural model was tested and revealed that response efficacy and attitude positively influenced organic food purchase intention; and attitude was the most important predictor of this intention. Knowing the influencing factors and differences of the target market from the socio-demographic characteristics will enable firms to create more specific selling points to market organic food to the right target markets, hence, contributing towards sustainability in the country.

## 1. Introduction

Globally, sustainability and the environmental issues are top of mind for many consumers when making decision about food choices. Organic food is commonly known as food produced from farming systems that avoid the utilization of artificial fertilizers and pesticides. Organic food provides health benefits with no chemical additives [1] and helps in minimizing negative impacts on the environment [2]. Nevertheless, the growth of organic food consumption is attributed to the increasing levels of concern for environmental and ecological welfare [3].

In Malaysia, the organic food market is relatively small compared to larger countries, such as the Unites States [4]. The value of Malaysia’s organic food production is expected to hit RM200 million by the year 2025, to satisfy local demand and the export market [5]. It will be challenging to convince consumers in the food market to change their eating habits and food choices towards a more sustainable food consumption pattern, as these are the central aspect of their lifestyles. As a result, consumers’ acceptance of or resistance toward organic food has become the focus of many academicians and industrial practitioners.

Socio-demographic factors, such as age, are important variables in consumer behavioral studies [6] as different age groups have different social, cultural, political and economic experiences. Although the literature on organic food is increasing, there is a lack of studies on organic food behavior of the Millennial generation [7]. Studies concerning organic food purchase intention among Millennials are needed as the organic food market will continue to grow and evolved [8]. Millennials are defined as those who are born between 1980s and the middle of 1990s [9,10].

According to Khidhir [11], by 2025, Millennials will make up nearly 75% of the global workforce. Millennials are more engaged with food and tend to be more health-conscious and eat healthier compared to other generations [12]. They are generally more environmentally conscious and understand better health problems in the communities; thus, they understand the importance of having food that is free from pesticides and other chemicals [13].

Similarly, Molinillo et al. [6] examined the antecedents of Millennials towards organic food purchase from the data collected from two distinct countries (i.e., Brazil and Spain) and showed that both health and social consciousness influenced their willingness to pay a price premium towards organic food. In Greece, Kamenidou et al. [13] revealed that organic food purchasing behavior was different from the five generational cohort surveyed in the study. Hence, it is important to understand whether there will be any significant differences between socio-demographic characteristics of Millennials in Malaysia and their intention to purchase organic food. The outcome of the study is essential when designing targeted marketing campaigns in the organic food market.

There are two objectives in this study. First, we aim to examine whether any significant differences exist between socio-demographic characteristics of Millennials and their intention to purchase organic food. Secondly, this study extends the Theory of Planned Behavior (TPB) by integrating variables from the Protection Motivation Theory (PMT) to investigate factors that influence Millennials’ intention to purchase organic food. PMT [14] is often used as theoretical basis for the study of personal protective behavior.

PMT is a social cognitive model that prioritizes relevant perceptions to predict health-related behaviors. The TPB-PMT integrated model has stronger explanatory power [15] and both theories have similarities that make them a good platform for theory integration [16,17]. Research in the context of organic food purchases among Millennials from multi-theoretical perspectives by combining TPB and PMT is a vital approach. The outcomes of this study will provide deep insights for any stakeholders who are involved in sustainable organic food chains to better segment and target the green market with more enduring and effective sales and marketing strategies. This will help to promote sustainable organic food consumption, thus, contributing towards sustainability goal in the country.

The remainder of this paper is structured as follows: In Section 2, we will discuss the literature review, research hypotheses and the research framework. In Section 3, we will explain the research design and methodology. This is followed by a report of the results in Section 4. In Section 5, we will provide discussion based on the results and compares with previous studies. Lastly, in Section 6, we conclude by discussing the theoretical contribution, practical implications, limitations and future research directions.

## 2. Literature Review, Hypotheses and Research Framework

### 2.1. Millennials

The term “millennial” is used all over the world; however, the age range varies between different studies. For instances, Weber [18] defined millennials as those born between 1980 and 2000, whereas Naderi and Van [19] defined millennials as those born between 1982 and 2000. In terms of behavior and consumption patterns, millennials are regarded as the most dynamic, informed and sensitized group [20].

Due to their size and purchasing power, which comprises a population of about two billion people worldwide who spend $200 billion per year [21], many researchers and companies from various industries started to pay more attention to studying their purchasing behavior. According to Weber ([18], p. 520), “academics see that this generation is more connected with others and with the society, as well as ready to contribute to the improvement of the world they live in”. Several studies suggested that millennials have favorable attitudes toward green consumption [19,22,23]; and the mixed results reported by Lian and Yoong [24] when investigating Millennials’ purchase intentions toward organic food, prompting calls for further research in emerging countries.

### 2.2. Theory of Planned Behavior

Ajzen and Madden’s [25] Theory of Planned Behavior (TPB) posits that one’s intention to engage in a particular behavior is due to positive attitudes toward such behavior (attitude), pressure from significant others (subjective norm) and perception of the ease or difficulty to perform the behaviors (perceived behavioral control). Past studies revealed that purchase intention of organic food is significantly influenced by three TPB’s core components: attitude, subjective norm and perceived behavioral control [26,27,28,29,30]. Consumers who have positive and favorable attitude tend to have higher intention to purchase organic food.

However, subjective norm and perceived behavioral control was found to be not significant predictors of intention in Wong et al. [31] as the purchasing behavior has not become a social norm among the community yet. PMT’s self-efficacy is said to be a better predictor in health-related studies as compared to TPB’s perceived behavioral control [32], though self-efficacy concept is similar to perceived behavioral control. Hence, self-efficacy is used in this study to avoid overlapping as perceived behavioral control is similar to self-efficacy.

### 2.3. Protection Motivation Theory

Protection Motivation Theory (PMT) proposed by Rogers [14] states that when an individual faces a threat, he will experience coping and a threat appraisal process for protecting himself. It highlights two important components; coping appraisal and threat appraisal. Coping appraisal highlights the coping responses to cope with a threat that includes self-efficacy and response efficacy, while threat appraisal involves the assessment of perceived threat level, includes perceived severity and perceived vulnerability. Perceived severity can be defined as an individual’s belief on how seriously a threat would affect one’s life [33], whereas perceived vulnerability refers to an individual’s belief on how susceptible one feels to a threat [34].

Perceived severity and perceived vulnerability were found to have positive relationship with purchase intention in past studies [35,36,37,38]. Nonetheless, Lwin et al. [39] found no significant relationship between perceived vulnerability and purchase intention as people tend to believe that they have lesser chance to encounter negative consequences. On the other hand, self-efficacy focuses on the beliefs that one is capable to perform the necessary course of action [40] and past studies revealed that self-efficacy significantly influence organic food purchase intention [26,41]. For response efficacy, one is considered to have high response efficacy when he believes that he can lessen the threat by engaging in a recommended behavior [26,42].

### 2.4. Hypothesis Development

In this study, twelve hypotheses are proposed:

#### 2.4.1. Gender and Intention

Consumer gender differences impact their organic food purchase intention [43]. Past studies revealed that females have more favorable attitudes towards organic food and higher intentions to purchase compared to males [44,45,46]. However, Munasinghe and Shantha [47] as well as Irandoust [48] found that gender had no significant effect on organic food purchase intention.

**H1:**  *There is a significant difference between gender of Millennials and their intention to purchase organic food.*

#### 2.4.2. Age and Intention

Past studies also revealed that age group is significantly and positively related to the organic food purchases [45], where older people are more likely to purchase organic food [49]. Nevertheless, results from Irandoust [48] showed that age does not influence intention to purchase organic food.

**H2:**  *There is a significant difference between age of Millennials and their intention to purchase organic food.*

#### 2.4.3. Marital Status and Intention

It is generally perceived that married household tend to purchase organic food, and this was supported by Dimitri and Dettmann [50]. In other words, single households are less likely to purchase organic food as compared to married households.

**H3:**  *There is a significant difference between marital status of Millennials and their intention to purchase organic food.*

#### 2.4.4. Education and Intention

Past studies discovered that educational level was significantly and positively related to organic food purchases [45], where consumers with a higher education level tend to purchase organic food [47,49]. Consumers with a university education level tend to have better awareness of health issues compared to people with lower level of education [33]. However, Irandoust [48] explained that educational level had no significant effect on intention to purchase organic food.

**H4:**  *There is a significant difference between educational level of Millennials and their intention to purchase organic food.*

#### 2.4.5. Occupation and Intention

Occupation represents an individual’s social identity and lifestyle, which may affect their organic food purchases [51]. Results by Yuan and Xiao [51] indicated that occupation had significant effect on purchase intention, where civil servants tended to purchase organic food compared to other occupational groups.

**H5:**  *There is a significant difference between occupation of Millennials and their intention to purchase organic food.*

#### 2.4.6. Income and Intention

Despite the increase in organic food purchases, income level has been identified as one of the driving forces to organic food purchases, and the correlation between income and organic food purchases has been varied [44]. Past studies revealed that income level as significantly and positively related to organic food purchase [48,49,52]. However, outcomes from Munasinghe and Shantha [47] and Omar et al. [45] found that income did not significantly and positively influence purchase intention.

**H6:**  *There is a significant difference between monthly income of Millennials and their intention to purchase organic food.*

#### 2.4.7. Ethnicity and Intention

Previous studies that investigated the role of ethnicity mostly relied on the U.S data [53]. Ethnicity tend to influence the likelihood of organic food purchases and in Dimitri and Dettmann [50], Asian households are more likely to purchase organic food. In this study, Malaysian consumers, who consist of Malay, Chinese, Indian and a proportion of other races, allow for a different analysis of the roles of ethnicity in affecting consumers’ intention to purchase organic food.

**H7:**  *There is a significant difference between ethnicity of Millennials and their intention to purchase organic food.*

#### 2.4.8. Prior Purchasing Experience and Intention

Prior purchasing experience influences consumer’s decision on whether to purchase organic food [54]. Thambiah et al. [55] found that Millennials’ experience and involvement with organic food is an important factor in their consumption decision.

**H8:**  *There is a significant difference between Millennials prior purchasing experience and their intention to purchase organic food.*

#### 2.4.9. Perceived Severity and Intention

Perceived severity denotes to the belief of an individual on how severe the damage that a particular threat can cause to that individual [33]. Past studies demonstrated that perceived severity positively affects organic food purchase intention as consumers perceived non-organic food to have more problems [36]. This is supported by Rainear and Christensen [38] who reported that perceived severity is a strong predictor of purchase intention. However, perceived severity was found to have no relationship with purchase intention in Park et al. [56] because the nature of sample used was a younger group of consumers who were less likely to perceive having health problems.

**H9:**  *Perceived severity influences Millennials purchase intention towards organic food positively.*

#### 2.4.10. Perceived Vulnerability and Intention

Perceived vulnerability assesses how personally susceptible an individual feels to a threat [34]. Past studies have shown different results in the relationship between perceived vulnerability as a predictor of purchase intention. Babazadeh et al. [37] discovered that when individuals have greater knowledge regarding vulnerability towards a behavior, consumers tend to engage in the recommended behavior. However, perceived vulnerability was found to have no relationship with purchase intention as consumers tend to believe they have higher chance to experience positive events than negative events, and youngers consumers tend to perceive to have fewer health problems than other age groups [35,39,56].

**H10:**  *Perceived vulnerability influences Millennials purchase intention towards organic food positively.*

#### 2.4.11. Response Efficacy and Intention

Response efficacy refers to the individual’s belief where the suggested behavior is helpful and effective in lessening a particular threat to the individual [34,57]. Past studies showed that response efficacy is a significant predictor of intention [26,58]. Consumers are more likely to have higher intention to engage in the behavior when they have adequate knowledge about the effectiveness of the behavior in lessening a threat or danger.

**H11:**  *Response efficacy influences Millennials purchase intention towards organic food positively.*

#### 2.4.12. Self-Efficacy and Intention

Self-efficacy can be described as an individual’s belief on his capability to perform necessary course of action to handle some potential situation [40]. It has been shown in the previous studies that self-efficacy significantly and positively affects purchase intention [26,41]. When consumers believe that purchasing organic food will help in enhancing their health and the environment, they are said to have high level of self-efficacy, and this leads to higher intentions to purchase organic food. 

In term of the Millennials, in Ramadhan et al. [59], perceived behavioral control, which has a similar concept to self-efficacy, had a significant and positive influence on Millennials’ intention to purchase organic food. However, self-efficacy was found to have no relationship with intention in Al-Swidi et al. [60]. The study found that consumers have lower purchase intention if they possess limited knowledge about the harmful events or recommended behavior and lower confidence in engaging the behavior.

**H12:**  *Self-efficacy influences Millennials purchase intention towards organic food positively.*

#### 2.4.13. Attitude and Intention

Attitude refers to an individual’s feeling, either positive or negative, about carrying out a particular behavior [61] (Nguyen et al., 2018). The relationship between attitude and intention has been widely tested, and the relationship has been proven to be significant in past studies [62,63,64]. However, research conducted by Juschten et al. [65] revealed that attitude is a very weak predictor of intention, possibly because attributes used to describe attitude were not specific enough. Patel et al. [66] explained that if Millennials develop a more positive attitude towards organic food, this will result in higher intention to purchase organic food.

**H13:**  *Attitude influences Millennials purchase intention towards organic food positively.*

#### 2.4.14. Subjective Norm and Intention

Subjective norm is the individual’s belief that he is motivated to perform the behavior if it is expected by the important others [61]. Subjective norm is a strong predictor of purchase intention [29,67] as consumers are more likely to comply with their closed one’s expectation. However, the subjective norm was found to have no significant effect on purchase intention in some past studies [31,68] where purchasing organic food has not become a social norm among consumers in the region yet. Similar non-significant results were found in Lavuri et al. [69] where the subjective norm did not impact Millennials’ purchase intention.

**H14:**  *Subjective norm influences Millennials purchase intention towards organic food positively.*

### 2.5. Research Framework

**Figure 1:**
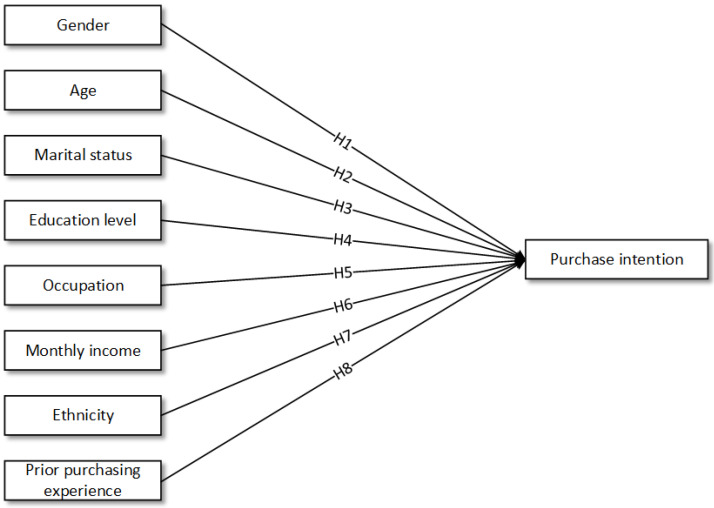
Socio-demographic characteristics and intention to purchase organic food (H1–H8).

**Figure 2:**
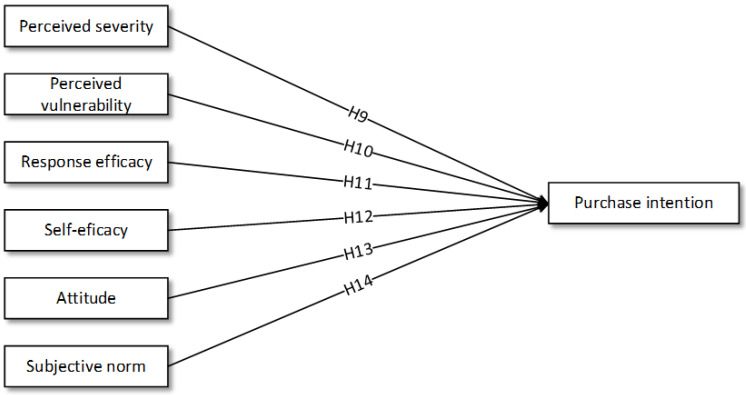
Multi-theoretical approach and purchase intention (H9–H14).

Based on previously cited theoretical and empirical literature, Figure 1 illustrates hypotheses H1 to H8 on differences between socio-demographic characteristics and intention to purchase organic food. Figure 2 proposes a research framework using multi theoretical approach on Millennials’ purchase intention towards organic food in Malaysia. Perceived severity, perceived vulnerability, self-efficacy and response efficacy were added to the TPB model.

## 3. Methods

Survey questionnaires were distributed face-to-face to target respondents in 2019. Following the definition of Millennials given by Dimock [9] and San et al. [10], the selected respondents (i.e., Millennials) were born between 1980s and the middle of 1990s and resided in Klang Valley, Malaysia. Since it was not possible to obtain a sampling frame from the local authorities, convenience sampling was employed. Target respondents were approached at both organic and non-organic food retail outlets to ensure there was a mixture of both organic and conventional food purchasers. Kline ([70] suggested that a sample size of 200 was appropriate for Structural Equation Modeling (SEM) analysis.

Participation in the study was voluntary, and a total of 214 questionnaires were collected. Several statistical analyses, such as descriptive analysis, independent sample T-test, Analysis of Variance (ANOVA) were conducted via SPSS. Descriptive statistics were used to describe the basic features of the data, such as the percentage and the average mean scores for each of the variables.

The independent sample T-test was used to compare the means between two groups. For instance, gender (female and male) and age groups (26–35 and 36–45 years). Whereas ANOVA was used to compare the means among three or more groups for other demographic characteristics except gender and age groups. In addition, PLS-SEM was applied to examine the structural relationship of the constructs proposed in the model. Both measurement and structural models were assessed in Section 4.

This study was based on quantitative research. The measurement scales of six constructs were adopted and adapted from existing validated scales. Items for perceived severity and perceived vulnerability were adapted from Rainear and Christensen [38], items for response efficacy were adapted from Ibrahim and Al-ajlouni [36], self-efficacy items were adapted from Wang et al. [35], attitude, subjective norm and purchase intention were adapted from Paul et al. [29]. All these scales were measured using a seven-point Likert agreement scale and is presented in Appendix A.

## 4. Results

The results of descriptive analysis, the independent sample T-test and analysis of variance (ANOVA) are first presented, followed by the measurement and structural model assessment. From the 214 samples collected, 52% of them were male, and 48% were female. Furthermore, 80% were between the ages of 26 and 35 years, 58% were single, 53.5% had a tertiary education; 32% worked as officers, and 42% earned RM5000 or more per month.

### 4.1. Gender, Age, Prior Experience and Independent Sample T-Test

Independent sample T-test was conducted to determine whether there is a significant difference between gender and purchase intention towards organic food (Hypothesis 1). Table 1 and Table 2 showed the frequency, percentage, average mean score of purchase intention towards organic food based on gender and results from the independent sample T-test by gender. A total of 112 males and 102 females participated in this study, with 52% and 48% of the sample size respectively. The results showed that the variances are not significantly different and purchase intention between gender is not significantly different (t_212_ = −0.629, *p* > 0.05). Therefore, it can be assumed that they are equal (F = 4.439, *p* < 0.05), and Hypothesis 1 was not supported.

Next, the frequency, percentage, average mean score of purchase intention towards organic food based on age group and results from the independent sample T-test by age group were presented in Table 1 and Table 2. The respondents consist of 172 who are from the 26–35 age group, while the rest of 42 respondents are from the 36–45 age group. The results showed that the variances are not significantly different and purchase intention between age group is not significantly different (t_212_ = −0.424, *p* > 0.05). Therefore, it can be assumed that they are equal (F = 0.070, *p* < 0.05) and Hypothesis 2 was not supported.

As for the prior experience versus intention, it was hypothesized in Hypothesis 8. As shown in Table 1, out of total of 214 respondents, 164 of them purchased organic food before whereas the rest of 50 do not have prior experience in purchasing organic food. In Table 2, the results revealed that the variances are significantly different and purchase intention towards organic food between prior experience is significantly different (t_212_ = 2.034, *p* < 0.05). Therefore, it can be assumed that they are not equal (F = 0.024, *p* > 0.05) and Hypothesis 8 was supported.

### 4.2. Other Socio-Demographic Characteristics and Analysis of Variance (ANOVA)

One-way Analysis of Variance (ANOVA) was applied to determine whether there were any significant differences between the socio-demographic characteristics of Millennials and their purchase intention towards organic food (H2–H7). One way ANOVA was used because each hypothesis only involves one independent variable, which has more than two categorical groups. Table 3 presented the ANOVA results for other socio-demographic characteristics in this study. Respondent profiles are as follows: 58% are single; 53.5% are Bachelor’s Degree holders; 32% are executive officers; 42% have monthly income above RM5001; and 71% are Chinese.

The ANOVA results indicated that H5 (F_7,206_ = 2.736; *p* = 0.010, < 0.05) and H6 (F_3,210_ = 3.834; *p* = 0.011, < 0.05) were supported, while H3 (F_2,211_ = 1.161; *p* = 0.315, > 0.05), H4 (F_4,209_ = 0.853; *p* = 0.493, > 0.05), H7 (F_2,211_ = 1.333; *p* = 0.266, > 0.05) were not supported. In other words, Malaysian Millennials’ occupation and monthly income were found to have significant difference on their intention to purchase organic food. In contrast, marital status, education level and ethnicity were found to have no significant differences on purchase intention towards organic food.

### 4.3. Assessment of Measurement Model

**Figure 3:**
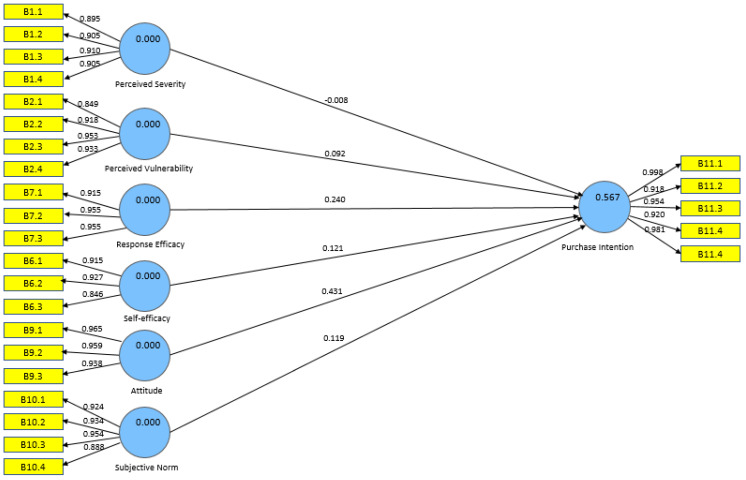
A multi-theoretical approach model.

Anderson and Gerbing’s two-step approach [71] was used to assess the multi-theoretical approach research model developed as shown in Figure 3. The measurement model was tested to investigate the reliability and validity of the instruments used followed by running the structural model to test the hypotheses.

#### 4.3.1. Convergent Validity

Convergent validity was tested to assess whether the measurement items of the construct were correlated through several criteria indicated by Hair et al. [72]. First, standardized factor loading estimates for each of the indicator should be 0.50 or above; second, an average variance extracted (AVE) of 0.50 or higher suggests adequate convergence; and third, the convergent validity is achieved if the composite reliability (CR) meets the cut-off criterion of 0.60. Factor loading estimates for all the measurements items were above the minimum point of 0.50; the CR values extended from 0.9247 to 0.9684, while AVE values ranged between 0.8038 and 0.9101 (Table 4). Hence, all measurements for the reliability as well as the validity of each construct were achieved.

#### 4.3.2. Discriminant Validity

Discriminant validity was examined using Fornell and Larcker [73] criterion and Heterotrait–Monotrait ratio of correlations (HTMT). Fornell and Larcker [73] suggested that a latent variable should interpret and explain better the variance on its own indicators, as compared to the variance of other latent variables. Table 5 indicates the square root of AVE of each construct is greater than the correlation with the others constructs. Therefore, all constructs obtained satisfactory result based on Fornell and Larckers’ Criterion; thus, discriminant validity has been ascertained.

### 4.4. Assessment of Structural Model

In order to test the significance of the relations, a Bootstrapping module was used. Then, the Predictive Relevance (Q^2^) and Effect Size (f^2^) were evaluated. The findings in Table 6 show that the predictive relevance (Q^2^) of purchase intention had a value of 0.3416, indicating that the model has sufficient predictive power as the Q^2^ values reported were significantly above zero. Next, to interpret the impact of f^2^ at the structural level, it has been suggested that the effect is large when f^2^ is 0.35, medium when f^2^ is 0.15 and small when f^2^ is 0.03 [74]. With this, the values of f^2^ reported in Table 7 range from 0.1187 t0 0.8458, which indicated that the constructs have medium to large effect in producing coefficient of determination score for purchase intention, Therefore, the model was accurate, and all the constructs were important for the general adjustment of the model.

### 4.5. Hypotheses Testing

According to the path coefficients and significance level as shown in Table 7, response efficacy (β = 0.2397, *p* < 0.000) and attitude (β = 0.4307, *p* < 0.000) significantly and positively influenced Millennials purchase intention towards organic food. Therefore, H11 and H13 were supported. Perceived severity (β = −0.0077, *p* < 0.000), perceived vulnerability (β = 0.0919, *p* < 0.000), self-efficacy (β = 0.1207, *p* <0.000) and subjective norm (β = 0.1188, *p* < 0.000) were not significant, which means that H9, H10, H12 and H14 were not supported. Further investigation discovered that attitude was the most important predictor of Millennials’ intention to purchase organic food, followed by response efficacy. Table 7 provides a summary of the results of hypotheses testing (H1–H14) in this study.

## 5. Discussion

In terms of socio-demographic indicators, occupation, monthly income and prior experience were found to have significant and positive influence towards Millennials consumers’ intention to purchase organic food, and the results wareere consistent with past studies [48,49,51,52,54]. The likelihood of purchasing organic food is increasing with income in a study conducted in the south of Sweden by Irandoust [48]. High income consumers were more likely to increase their purchase of organic food products.

The remaining socio-demographic characteristics: gender, age, marital status, educational level and ethnicity were found to have no significant relationship with Millennial’s purchase intention towards organic food. This was in line with the results reported from Munasinghe and Shantha [47] and Irandoust [48]. Other possible explanations on the insignificant results for H1 (Gender), H2 (Age), H3 (Marital Status), H4 (Education Level) and H7 (Ethnicity) is that Millennials, regardless of their gender, age group, marital status, educational level and ethnicity, appear to think similarly in terms of purchase intention across these demographic factors.

Millennials were born in the time of economic prosperity (in the 1980s and 1990s) and were exposed to a much open concept of consumerism, technological advancement, better standard of living and higher educational attainment. Additionally, many of them have similar outlooks and exposure via mass and social media on issues, such as the environment, sustainability, circular economy and organic food consumption. They were exposed at an early age to these concepts regardless of their background. This could be a possible reason why certain demographic factors did not yield significant results. In summary, for the first part of the analysis on the social-demographic characteristics, H1, H2, H3, H4 and H7 were not supported, while H5, H6 and H8 were supported.

Next, H9 and H10 were not supported as the results showed that perceived severity and perceived vulnerability did not significantly influence Millennials’ intention to purchase organic food. This was in line with past studies that revealed perceived severity and perceived vulnerability did not have the direct relationship with purchase intention [35,39,56]. For instance, Wang et al. [35] found that farmers perceived that susceptibility and severity of threats caused by water deterioration influenced environmental intention through the mediating effect of subjective norm and attitude toward adopting pro-environmental behavior.

In this study, the results showed that Millennials, the younger consumers, are likely to perceive to have lower chance to experience negative events and less likely to be affected by threats. With this, the mediating variable may need to be explored in future studies in the similar context. Millennials seem to perceive that the situation in terms of environmental degradation, climate change, effect on planet earth is not severe enough to affect their decision to purchase more organic food. These issues are important to them; however, their perception seems to be that these doomsday predictions of the future are not urgent and severe enough for them to take drastic measures to change their buying behavior towards consuming organic food.

As for H11, the results indicated that there was significant positive effect of response efficacy towards purchase intention. This was consistent with past studies by Verkoeyenand Nepal [26] and Meso et al. [58], who applied PMT in studies of intended adaptation to coral bleaching and information security training, respectively. In the study of Verkoeyen and Nepal [26], although the research was not in the context of organic food purchase, PMT was able to explain between 12.8% and 47.7% of the variance in adaptation intentions, with response efficacy and self-efficacy consistently emerging as the strongest significant predictors.

Therefore, results obtained from the present study showed that Millennials believed that purchasing organic food was helpful and effective in lessening threats to the individuals. Millennials tended to have more knowledge about organic food [75] and effectiveness of purchasing organic food in lessening the particular threat, and this led to higher intention to purchase organic food. Hence, H11 was supported.

Hypothesis H12 was not supported as self-efficacy was found to have no relationship with purchase intention. This result was in accordance to research by Al-Swidi et al. [60] who applied TPB and reported that the effect of perceived behavioral control on intention to buy organic food was not statistically supported based on the data collected from the academic staffs and students of two universities in southern Punjab, Pakistan. According to the result in this study, Millennials tend to believe that they are not capable to purchasie organic food. One of the reasons could be because they have limited knowledge about the harmful events of not purchasing organic food and less confident in engaging in the behavior, thus, leading to a lower intention to purchase organic food.

Attitude was found to have significant and positive relationship with Millennials’ purchase intention towards organic food. This was in line with past studies [62,63,64]. Therefore, H13 was supported. The results showed that Millennials are inclined to develop a more favorable and positive attitude towards organic food, thus, leading to a higher intention to purchase organic food. It was interesting to note that attitude was a significant predictor of organic food purchase intention, regardless of whether the respondents were from the non-Millennials [62,63,64] or Millennials groups that were surveyed in this study. Hypothesis H14 was not supported as subjective norm had no significant relationship with purchase intention.

Similar to the results shown in the past [31,68,69], the subjective norm was found to have no impact on Millennials’ purchase intention. This could possibly because purchasing organic food has not yet become a social norm among Millennials in Malaysia. As a result, they were not motivated to purchase organic food as it was not widely expected by their important others to engage in the behavior. On the other hand, the result of the study conducted by Al-Swidi et al. [60] in Pakistan showed that subjective norms significantly moderate the relationship between attitudes and buying intention as well as between perceived behavioral control and buying intention. These warrants future research attention to consider modeling subjective norm as a moderator in the structural model instead of a direct predictor of behavioral intention.

## 6. Conclusions

The purpose of this study was to investigate the influencing factors of consumer purchase intentions towards organic food from the perspective of the Millennials generation in Malaysia. The significant differences between the socio-demographic characteristics of Millennials and their intentions were examined. In terms of the theoretical contribution, instead of applying a single model, such as TPB, to understand the organic food purchase intention of Millennials, a multi-theoretical approach by including variables from PMT together with variables from TPB could provide a fuller understanding of market information from the domain of organic food purchase.

A number of past researchers argued that TPB places too much emphasis on the rationality of the human subject as an actor and inadequately accounts for other factors (i.e., psychological/social) that played equally crucial roles in determining behavior. TPB will be a better predicting model if other factors are incorporated in the study [76].

The results concluded that there was no significant difference between gender group of Millennials and their intention to purchase organic food; while occupation, monthly income and prior experience were found to have differences regarding this intention. Response efficacy and attitude positively affected Millennials’ purchase intention towards organic food, and attitude was the most important predictor of purchase intention. From the practical implications, understanding similarities and differences of the target market from the socio-demographic characteristics will allow firms to create more specific selling points to promote sustainable food consumption, thus, moving closer towards sustainability goals in the country.

The results of the present study demonstrated that there is a necessity to influence Millennials’ perception that their action to purchase organic food will make a difference in reducing or eliminating the perceived threat from environmental and ecological problems. It will be timely and important to cultivate favorable and positive attitudes among Millennials in the advertising and promotion campaign as findings showed that a more positive attitude will further reinforce their purchasing intentions.

The outcome of the present study will provide useful information to organic food retailers or marketers who are seeking to improve their sales, achieve continuous business growth and sustain their presence in the market. Knowing the influencing factors towards purchase intention, both existing and potential market players in Malaysia could plan and develop effective targeting and positioning strategies to increase market demand on the organic food products.

Lastly, due to the limitation of the cross-sectional study, a longitudinal approach could be employed to collect data in future studies to determine the variability of patterns over time. Investigations into specific organic foods, such as organic meat or organic vegetables, could be considered in the future in addition to understanding the market responses regarding the general aspects of organic food in the market.

## Figures and Tables

**Table 1:** The results of the independent sample T-test and average mean scores.

		Frequency (N)	Percentage (%)	Average Mean	*p*-Value	Findings	Hypothesis
Gender	Male	112	52	4.8875	0.530	Not significant	H1 is not supported
	Female	102	48	4.9765			
Age	26–35	172	80	4.9151	0.672	Not significant	H2 is not supported
	36–45	42	20	4.9905			
Prior Experience	Yes	164	77	1.0565	0.043	Significant	H8 is supported
	No	50	23	0.9087			

Note: *p* < 0.05.

**Table 2:** Levene’s test for equality of variances and T-test for equality of means.

	Levene’s Test for Equality of Variances		T-Test for Equality of Means		
	F	Sig.	t	df	Sig. (2-Tailed)
Gender	4.439	0.036	−0.629	212	0.530
Age	0.070	0.791	−0.424	212	0.672
Prior Experience	0.024	0.877	2.034	212	0.043

Note: *p* < 0.05.

**Table 3:** ANOVA results for other socio-demographic characteristics.

Socio-Demographic Characteristics	Frequency (N)	Percentage (%)	df (Between Groups)	df (Within Groups)	F	*p*-Value	Findings	Hypothesis
**Marital Status**			2	211	1.161	0.315	Not significant	H3 is not supported
Single	124	58						
Married	85	40						
Divorced	5	2						
**Education Level**			4	209	0.853	0.493	Not significant	H4 is not supported
Secondary school	30	14						
Certificate/Diploma	32	15						
Bachelor’s Degree	115	53.5						
Postgraduate Degree	37	17.5						
**Occupation**			7	206	2.736	0.01	Significant	H5 is supported
Self-employed	60	28						
Professional	47	22						
Manager/Senior	19	9						
Executive Officer	69	32						
Housewife	4	2						
Student	7	3						
Unemployed	2	1						
Others	6	3						
**Monthly Income**			3	210	3.834	0.011	Significant	H6 is supported
RM1500 and below	20	9						
RM1501-RM3500	27	13						
RM3501-RM5000	76	36						
RM5001 and above	91	42						
**Ethnicity**			2	211	1.333	0.266	Not significant	H7 is not supported
Malay	47	22						
Chinese	152	71						
Indian	15	7						

Note: *p* < 0.05.

**Table 4:** Indicator Loadings, Composite Reliability and AVE.

Constructs	Items	Loadings	Composite Reliability	AVE
Perceived Severity (PS)	PS1	0.8952	0.9469	0.8186
	PS2	0.9052		
	PS3	0.9099		
	PS4	0.9047		
Perceived Vulnerability (PV)	PV1	0.8489	0.9529	0.8353
	PV2	0.9181		
	PV3	0.9528		
	PV4	0.9325		
Response Efficacy (RE)	RE1	0.9152	0.9591	0.8867
	RE2	0.9541		
	RE3	0.9551		
Self-efficacy (SE)	SE1	0.9146	0.9247	0.8038
	SE2	0.9268		
	SE3	0.8462		
Attitude (AT)	AT1	0.9645	0.9681	0.9101
	AT2	0.9592		
	AT3	0.9380		
Subjective Norm (SN)	SN1	0.9236	0.9597	0.8561
	SN2	0.9340		
	SN3	0.9542		
	SN4	0.8880		
Purchase Intention (PI)	PI1	0.9277	0.9684	0.8597
	PI2	0.9182		
	PI3	0.9200		
	PI4	0.9390		
	PI5	0.9309		

**Table 5:** Fornell–Larcker’s Criterion result.

	AT	PS	PV	PI	RE	SE	SN
AT	**0.9540**						
PS	0.3593	**0.9038**					
PV	0.3381	0.6904	**0.9139**				
PI	0.6769	0.3257	0.3434	**0.9272**			
RE	0.5006	0.2516	0.2193	0.5694	**0.9416**		
SE	0.4278	0.3589	0.3597	0.4833	0.4320	**0.8965**	
SN	0.3902	0.0978	0.1284	0.4316	0.3691	0.3749	**0.9253**

Legend: PS = Perceived Severity; PV = Perceived Vulnerability; RE = Response Efficacy; SE = Self-Efficacy; AT = Attitude; SN = Subjective Norm; PI = Purchase Intention.

**Table 6:** Values of the Indicators of Predictive Relevance (Q^2^) and Effect Size (f^2^).

Constructs	Q^2^	f^2^
Perceived Severity		0.1187
Perceived Vulnerability		0.1337
Response Efficacy		0.4798
Self-efficacy		0.3047
Attitude		0.8458
Subjective Norm		0.2289
Purchase Intention	0.3416	

**Table 7:** Summary of the results for hypotheses H1 to H14.

Hypothesis	Relationship	Path Coefficient	*p*-Value	Results
H1	Gender → Purchase Intention	-	0.530	Not supported
H2	Age → Purchase Intention	-	0.672	Not supported
H3	Marital Status → Purchase Intention	-	0.315	Not supported
H4	Education Level → Purchase Intention	-	0.493	Not supported
H5	Occupation → Purchase Intention	-	0.010	Supported
H6	Monthly Income → Purchase Intention	-	0.011	Supported
H7	Ethnicity → Purchase Intention	-	0.266	Not supported
H8	Prior Experience → Purchase Intention	-	0.043	Supported
H9	Perceived Severity → Purchase Intention	−0.0077	0.898	Not supported
H10	Perceived Vulnerability → Purchase Intention	0.0919	0.172	Not supported
H11	Response Efficacy → Purchase Intention	0.2397	0.000	Supported
H12	Self-efficacy → Purchase Intention	0.1207	0.086	Not supported
H13	Attitude → Purchase Intention	0.4307	0.000	Supported
H14	Subjective Norm → Purchase Intention	0.1188	0.074	Not supported

Note: - not tested in Figure 3.

## Data Availability

The data are available from the corresponding author.

## Appendix A

**Table A1:** Research Instrument.

Construct	Items
***Perceived severity***	Climate change is a serious issue.
	Climate change will have negative consequences on this planet.
	The negative impact of climate change is severe.
	The thought of climate change scares me.
***Perceived vulnerability***	Climate change can negatively affect me.
	I will experience the negative effects of climate change in my lifetime.
	I am vulnerable to the negative effects of climate change.
	My chances of being affected negatively by climate change are high.
***Response efficacy***	I am sure that purchasing organic food is effective in preventing negative environment effects.
	I am sure that purchasing organic food will help to prevent depletion of the scarce resources.
	I am sure that purchasing organic food will help to prevent threat to my well-being and the well-being of society.
***Self-efficacy***	It is easy for me to be involved in purchases of organic food.
	If I wanted to, I could easily be involved in purchases of organic food.
	It is mostly up to me whether I would like to be involved in purchases of organic food.
***Attitude***	I like the idea of purchasing organic food.
	Purchasing organic food is a good idea.
	I have a favourable attitude toward purchasing organic food.
***Subjective norm***	Most people who are important to me would want me to purchase organic food.
	People whose opinions I value would prefer that I purchase organic food.
	My friend’s positive opinion influences me to purchase organic food.
***Purchase intention***	I will consider buying organic food because they are less polluting in coming times.
	I will consider switching to organic food for ecological reasons.
	I plan to spend more on organic food rather than non-organic food.
	I expect to purchase organic food in the future because of its positive environmental contribution.
	I definitely want to purchase organic food in the near future.
